# Distinctive effects of executive dysfunction and loss of learning/memory abilities on resting-state brain activity

**DOI:** 10.1038/s41598-022-07202-7

**Published:** 2022-03-02

**Authors:** Hideyuki Hoshi, Yoko Hirata, Momoko Kobayashi, Yuki Sakamoto, Keisuke Fukasawa, Sayuri Ichikawa, Jesús Poza, Víctor Rodríguez-González, Carlos Gómez, Yoshihito Shigihara

**Affiliations:** 1 Precision Medicine Centre, Hokuto Hospital, Kisen-7-5 Inadacho, Obihiro, Hokkaido 080-0833 Japan; 2 Department of Neurosurgery, Kumagaya General Hospital, Kumagaya, 360‑8567 Japan; 3 Precision Medicine Centre, Kumagaya General Hospital, Kumagaya, 360‑8567 Japan; 4 Clinical Laboratory, Kumagaya General Hospital, Kumagaya, 360‑8567 Japan; 5 Biomedical Engineering Group, Higher Technical School of Telecommunications Engineering, University of Valladolid, 47011 Valladolid, Castilla y León Spain; 6 Centro de Investigación Biomédica en Red en Bioingeniería, Biomateriales Y Nanomedicina, (CIBER-BBN), 47011 Valladolid, Castilla y León Spain; 7 Instituto de Investigación en Matemáticas (IMUVA), University of Valladolid, 47011 Valladolid, Castilla y León Spain

**Keywords:** Diagnostic markers, Dementia, Cognitive ageing, Cognitive neuroscience, Neuronal physiology

## Abstract

Dementia is a syndrome characterised by cognitive impairments, with a loss of learning/memory abilities at the earlier stages and executive dysfunction at the later stages. However, recent studies have suggested that impairments in both learning/memory abilities and executive functioning might co-exist. Cognitive impairments have been primarily evaluated using neuropsychological assessments, such as the Mini-Mental State Examination (MMSE). Recently, neuroimaging techniques such as magnetoencephalography (MEG), which assess changes in resting-state brain activity, have also been used as biomarkers for cognitive impairment. However, it is unclear whether these changes reflect dysfunction in executive function as well as learning and memory. In this study, parameters from the MEG for brain activity, MMSE for learning/memory, and Frontal Assessment Battery (FAB) for executive function were compared within 207 individuals. Three MEG parameters were used as representatives of resting-state brain activity: median frequency, individual alpha frequency, and Shannon’s spectral entropy. Regression analysis showed that median frequency was predicted by both the MMSE and FAB scores, while individual alpha frequency and Shannon’s spectral entropy were predicted by MMSE and FAB scores, respectively. Our results indicate that MEG spectral parameters reflect both learning/memory and executive functions, supporting the utility of MEG as a biomarker of cognitive impairment.

## Introduction

Dementia is a syndrome characterised by progressive deterioration of cognitive functioning due to various brain pathologies. The most common form of dementia is Alzheimer's disease (AD), accounting for approximately 60–80% of all dementia cases^1^. Its effects include impaired learning/memory, language, problem-solving, attention, and behaviour. The loss of learning/memory abilities is often the chief complaint of patients with early-stage dementia^2^. These functions are served by the temporal cortex, where pathological change initially starts^2^. The Mini-Mental State Examination (MMSE), which is the most commonly used assessment tool for dementia screening^3^, primarily evaluates learning/memory performance^4^. Executive function is another sub-domain of cognitive function that includes attention, inhibition, working memory, interference control, and cognitive flexibility^5^, which is served by the prefrontal cortex^6^. It is believed that executive function is not affected until the later stages of AD^2^; however, recent studies have revealed that it can manifest in the earlier stages^7^.

Although learning/memory and executive functions interact with each other, they are independent to some extent^8^ and are served by different brain regions. Hence, the tools used to measure both are also different: the MMSE for learning/memory^4^ and the Frontal Assessment Battery (FAB) for executive function^9^. The independence of these sub-domains of cognitive function is reflected by the fact that the MMSE is not sensitive to frontal-executive dysfunction^10^, and fails to detect mild cognitive impairment (MCI) in approximately a third of individuals^11^. Moreover, although both neuropsychological assessments are validated and easy to use in clinical settings, they have inherent limitations, such as skill dependence, subjectivity, ceiling effects, and practice effects.

To overcome these limitations, neuroimaging techniques such as electroencephalography (EEG) and magnetoencephalography (MEG) have come into use. EEG and MEG are able to evaluate resting-state brain activity in terms of neural oscillatory activity, whose changes are associated with cognitive impairment^12–16^. The changes are represented by (1) enhanced low-frequency oscillatory activity accompanied by attenuated high-frequency oscillatory activity, (2) slowing down of the alpha peak frequency, (3) less prominent alpha oscillations, and (4) loss of diversity of neural oscillatory components^17–19^. Previous studies have shown that these characteristics vary significantly between healthy individuals and individuals with MCI or AD^19,20^; hence, they can be considered potential biomarkers of cognitive impairment. However, previous studies have not evaluated the performance of these biomarkers in different sub-domains of cognitive impairment, which is crucial considering that dementia is defined by cognitive symptoms rather than a causative pathology. Therefore, in this study, we retrospectively analysed whether the resting-state brain activity acquired using MEG can assess learning/memory and executive functions-assessed using MMSE and FAB scores. Our analyses aimed to determine whether MEG biomarkers are sensitive to learning/memory and executive function.

## Results

### Descriptive statistics

**Figure 1:**
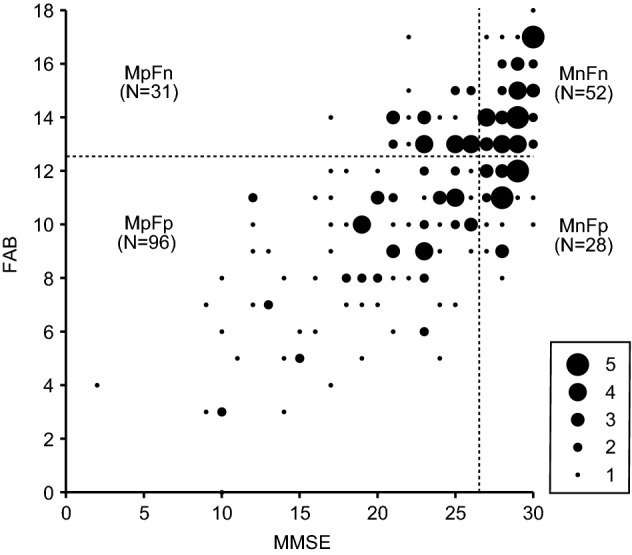
Relationship between Mini-Mental State Examination (MMSE) and Frontal Assessment Battery (FAB) scores. The sizes of the circles indicate the number of individuals who obtained a given score, and figures in the brackets represent the number of individuals who belong to each group. Broken lines indicate cut-off scores. Mn, MMSE-negative; Fn, FAB-negative; Mp, MMSE-positive; Fp, FAB-positive.

Clinical data from 207 individuals were retrospectively analysed. The average MMSE and FAB scores were 23.4 ± 5.6 (mean ± standard deviation, SD) (ranging from 2 to 30) and 11.2 ± 3.3 (mean ± SD) (ranging from 0 to 18), respectively. All individuals were divided into four groups according to their scores. Ninety-six individuals scored lower than the cut-off values for both MMSE (26 or below) and FAB (12 or below) (MMSE positive and FAB positive, MpFp), and 52 scored higher than those cut-offs (MMSE negative and FAB negative, MnFn). Thirty-one individuals scored lower than the MMSE cut-off but higher than the FAB cut-off (MpFn), and 28 scored higher than the MMSE cut-off but lower than the FAB cut-off (MnFp). This means that MMSE and FAB scores were mismatched for 59 (31 + 28) individuals (see Fig. 1). The detailed descriptive statistics and group-level differences are described in Supplementary information (Descriptive Statistics).

### Correlation analysis

**Figure 2:**
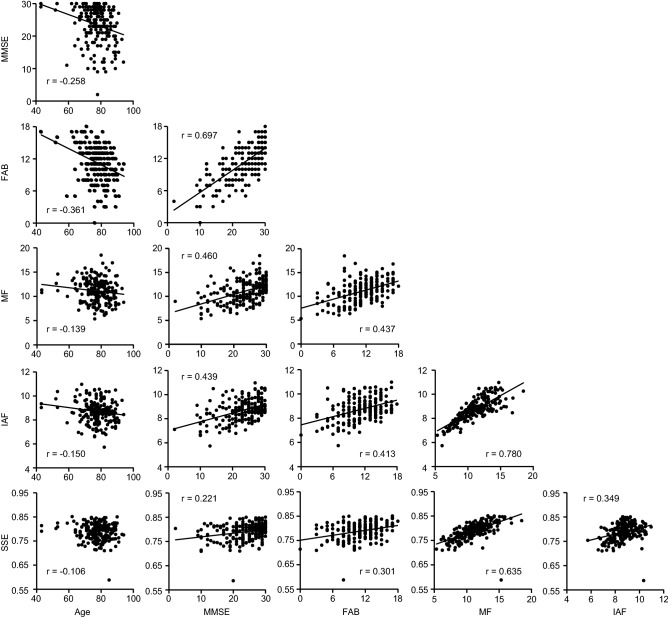
Correlations between age, neuropsychological assessment scores, and MEG spectral parameters. All pairs show significant correlations, except for age and SSE. Regression lines are added for significant correlations. MF and IAF are expressed in Hertz, and age in years. MMSE, Mini-Mental State Examination; FAB, Frontal Assessment Battery; MF, Median Frequency; IAF, Individual Alpha Frequency; SSE, Shannon's Spectral Entropy; r, averaged correlation coefficient across bootstrap iterations.

Three MEG spectral parameters were introduced to quantify the properties of resting-state brain activity: median frequency (MF), individual alpha frequency (IAF), and Shannon's spectral entropy (SSE). They were compared with age and MMSE and FAB scores using bootstrap correlation analysis. Age was negatively correlated with both neuropsychological assessment scores (MMSE: *r* = − 0.258, *p* < 0.001 and FAB: *r* = − 0.361, *p* < 0.001) and two MEG spectral parameters (MF: *r* = − 0.139, *p* = 0.013 and IAF: *r* = − 0.150, *p* = 0.015), except for SSE (*r* = − 0.106, *p* = 0.059) (Table 1 and Fig. 2). Age showed greater correlations with the MMSE and FAB scores than with the MEG spectral parameters. All MMSE and FAB scores and MEG spectral parameters correlated positively with each other. The MMSE and FAB scores were correlated positively (*r* = 0.697, *p* < 0.001).

**Table 1:** Correlation matrix between age, neuropsychological assessment scores, and MEG spectral parameters.

	AGE		MMSE		FAB		MF		IAF	
	*r*	*p* (FDR)	*r*	*p* (FDR)	*r*	*p* (FDR)	*r*	*p* (FDR)	*r*	*p* (FDR)
MMSE	− 0.258	< 0.001*								
FAB	− 0.361	< 0.001*	0.697	< 0.001*						
MF	− 0.139	0.013*	0.460	< 0.001*	0.437	< 0.001*				
IAF	− 0.150	0.015*	0.439	< 0.001*	0.413	< 0.001*	0.780	< 0.001*		
SSE	− 0.106	0.059	0.221	0.001*	0.301	< 0.001*	0.635	< 0.001*	0.349	< 0.001*

*MMSE*, Mini-Mental State Examination; *FAB*, Frontal Assessment Battery; *MF*, Median Frequency; *IAF*, Individual Alpha Frequency; *SSE*, Shannon’s Spectral Entropy; *r*, averaged correlation coefficient across bootstrap iterations; *p* (FDR), *p*-values of bootstrapping statistics with FDR correction. An asterisk (*) indicates statistically significant correlations after FDR correction.

### Regression analyses

**Figure 3:**
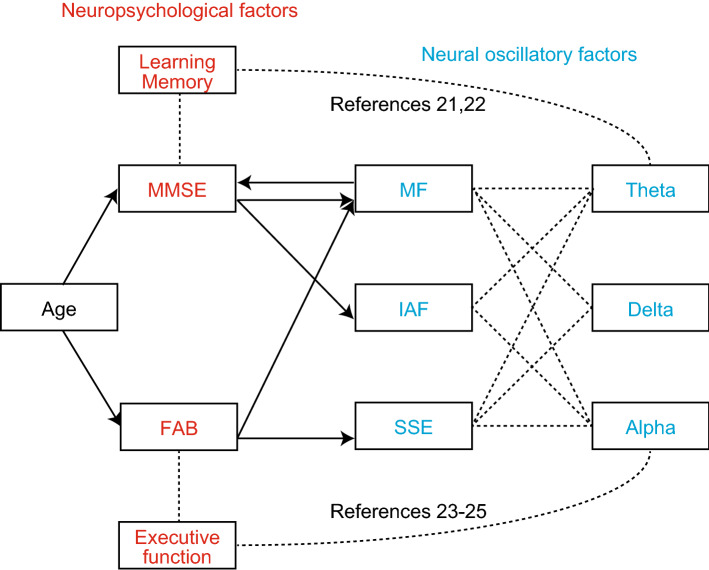
Associations between age, neuropsychological assessment scores, and MEG spectral parameters. Arrows indicate significant directional influence revealed by regression analysis. Dashed lines indicate associations described in previous studies^21–25^. MMSE, Mini-Mental State Examination; FAB, Frontal Assessment Battery; MF, Median Frequency; IAF, Individual Alpha Frequency; SSE, Shannon’s Spectral Entropy.

Directional relationships between MEG spectral parameters and neuropsychological assessments were studied using a linear mixed-effect model (LMEM), where biases from individuals’ age and sex were considered as nuisance variables. The LMEM analysis was performed bi-directionally, with either MEG spectral parameters or neuropsychological assessments used as response variables. MF was predicted by both MMSE (*β* = 0.298, *p* = 0.005) and FAB (*β* = 0.214, *p* = 0.046) scores. IAF was predicted only by MMSE score (*β* = 0.291, *p* = 0.005), but not FAB. SSE was predicted only by FAB scores (*β* = 0.282, *p* = 0.014) (Table 2 and Fig. 3). MMSE scores were predicted by age (*β* = − 0.190, *p* = 0.009) and MF (*β* = 0.410, *p* = 0.012), but not by IAF or SSE. FAB scores were predicted only by age (*β* = − 0.301, *p* < 0.001), but not by any of the MEG spectral parameters (Table 3 and Fig. 3). Two additional analyses were performed to further examine these results. For the first analysis, we applied an analogous protocol on the frontal and temporal sensors of interest (SOIs) (instead of all 160 sensors) as executive function (FAB) and learning/memory (MMSE) depend on these areas^2,6^. The results are described in Supplementary information (Regression analysis with MEG sensors of interest). For the second analysis, we considered only the healthy individuals’ dataset, as cognitive decline due to healthy ageing and pathological change could lead to different relationships between MEG spectral parameters and neuropsychological assesment scores; hence, we used the dataset of 34 individuals with healthy ageing (a subset of the present dataset) in the control analysis. The results are shown in the Supplementary information (Regression analysis with individuals with healthy ageing).

**Table 2:** Results of LMEM examining the effects of age and neuropsychological assessment scores on MEG spectral parameters.

	MF				IAF				SSE			
	β	*SE*	*t*	*p* (FDR)	β	*SE*	*t*	*p* (FDR)	β	*SE*	*t*	*p* (FDR)
Intercept	− 0.019	0.113	− 0.169	0.992	− 0.022	0.097	− 0.226	0.992	− 0.001	0.081	− 0.010	0.992
Age	0.006	0.067	0.089	0.992	− 0.014	0.096	− 0.148	0.992	− 0.008	0.097	− 0.080	0.992
MMSE	0.298	0.084	3.555	0.005*	0.291	0.085	3.421	0.005*	0.020	0.092	0.214	0.992
FAB	0.214	0.087	2.446	0.046*	0.185	0.089	2.094	0.090	0.282	0.095	2.955	0.014*

*MMSE*, Mini-Mental State Examination; *FAB*, Frontal Assessment Battery; *MF*, Median Frequency; *IAF*, Individual Alpha Frequency; *SSE*, Shannon’s Spectral Entropy; *β* standardised estimated coefficient of the predictor; *t*, t-values for testing the null hypothesis that the coefficients are equal to zero; *SE*, standard error of estimated coefficient; *p* (FDR), *p*-values of t-test with FDR correction. An asterisk (*) indicates terms that significantly contributed to the model.

**Table 3:** Results of LMEM examining the effects of age and MEG spectral parameters on neuropsychological assessment scores.

	MMSE				FAB			
	β	*SE*	*t*	*p* (FDR)	β	*SE*	*t*	*p* (FDR)
Intercept	0.013	0.061	0.209	0.928	< 0.001	0.071	< 0.001	1.000
Age	− 0.190	0.060	− 3.168	0.009*	− 0.301	0.059	− 5.107	< 0.001*
MF	0.410	0.139	2.954	0.012*	0.223	0.121	1.846	0.166
IAF	0.132	0.100	1.320	0.301	0.168	0.098	1.714	0.176
SSE	− 0.101	0.080	− 1.255	0.301	0.068	0.078	0.863	0.486

*MMSE*, Mini-Mental State Examination; *FAB*, Frontal Assessment Battery; *MF*, Median Frequency; *IAF*, Individual Alpha Frequency; *SSE*, Shannon’s Spectral Entropy; *β*, standardised estimated coefficient of the predictor; *t*, t-values for testing the null hypothesis that the coefficients are equal to zero; *SE*, standard error of estimated coefficient; *p* (FDR), *p*-values of t-test with FDR correction. An asterisk (*) indicates terms that significantly contributed to the model.

## Discussion

This study showed that both sub-domains of cognitive functions—learning/memory and executive function—were associated with MEG spectral parameters. MF was sensitive to both sub-domains, while IAF and SSE were only sensitive to learning/memory and executive function, respectively.

Dementia is a medical condition that is not defined by a causative pathology, but by symptoms that reflect deteriorating cognitive functions such as learning, memory, and executive function. It is believed that while learning/memory is affected in the earlier stages of AD, executive function is not. A previous study reported that decline in episodic memory occurs 7 years before the diagnosis of dementia, while decline in executive function occurs 2–3 years before diagnosis at group-level^26^. The early onset of decline in memory is supported by the fact that loss of learning/memory ability is served by atrophy observed in the temporal lobe, which changes during the earlier stages of AD. However, recent studies have shown that a deficit in executive function can also occur during the early stages of AD^7^. Particularly, pathological changes in the prefrontal cortex can manifest early due to reduced white matter tract integrity rather than cortical atrophy^27^. This aligns with our findings of 28 individuals scoring lower than the FAB threshold, but not the MMSE threshold (MnFp). Moreover, these results imply that executive dysfunction can precede the dysfunction in learning/memory at the individual level. In contrast, 31 individuals were MpFn, implying that learning/memory (*i.e.* MMSE) and executive function (*i.e.* FAB) are independent to some extent, and dysfunctions in both sub-domains are not always simultaneous. Therefore, to effectively screen patients with cognitive impairment at an early stage, cognitive dysfunction should be evaluated at least in two axes: learning/memory and executive function. This raises the question of how these two sub-domains of cognition are associated with MEG biomarkers.

In the present study, we focused on three MEG spectral parameters (*i.e.* MF, IAF, and SSE) as potential biomarkers that are sensitive to cognitive impairment^12–16^. These parameters have been clinically used at Kumagaya General Hospital since 2019. Throughout the analysis, we also considered the influence of age, as it is a major risk factor for dementia^28^ and influences MEG spectral parameters across the lifespan^29^ (Table 1). Correlation analyses showed that both neuropsychological assessment scores correlated with age. Although MEG spectral parameters, except for SSE, also correlated with age, their correlation coefficients were smaller than those of neuropsychological assessments. Previous studies have shown that MEG signals can be affected by various biological conditions that are independent of age, such as fatigue^30^, endocrinological conditions^31^, and appetite^32^. Therefore, it is understandable that age is more weakly correlated with the MEG spectral parameters than with neuropsychological assesment scores. Correlation analyses also showed that neuropsychological assessment scores were associated with MEG spectral parameters, aligning with previous findings of associations between spontaneous neural oscillatory activity (*i.e.* resting-state brain activity) and cognitive impairment^12–16^.

Regression analyses showed that MF was predicted by both MMSE and FAB, while IAF and SSE were predicted by MMSE and FAB, respectively (Table 2 and Fig. 3). These results suggest that MF is sensitive to both sub-domains (learning/memory and executive function). Both MF and IAF represent the spectral power balance between low- and high-frequency neural oscillatory activity: MF reflects the power balance in a wide frequency range, while IAF mainly reflects the theta and alpha bands. They decrease due to increased slow neural oscillatory activity, such as delta and theta oscillatory activities. A previous study showed that an increase in theta oscillations was associated with decreased hippocampal volume^33^, whose major functions are learning/memory^21^. Other studies have shown that theta oscillations are associated with memory^22,34^. Thus, it is presumable that MF and IAF are associated with MMSE scores (*i.e.* learning/memory). Delta oscillations are associated with the cholinergic neuronal activity, whose single source is the nucleus basalis of Meynert in the basal forebrain, which projects both frontal and temporal cortices^35^, modulating both learning/memory^36^ and executive function^37^. As AD causes loss of cholinergic neurons in the nucleus basalis of Meynert^38^, and deafferentation of the cortex from cholinergic inputs increases cortical delta oscillations^39^, it is possible that changes in MF were associated with both learning/memory (*i.e.* temporal lobe) and executive functions (*i.e.* frontal lobe); although, IAF was not associated with executive functions.

The SSE is another spectral parameter that quantifies the irregularity of the normalised power spectral density (PSDn) of resting-state neural oscillatory activity. The SSE increases to 1.0 when oscillatory power is equally distributed across the whole frequency spectrum (*i.e.* white noise) and decreases to 0 when some specific frequencies are prominent. Previous studies have revealed that patients with AD have lower SSE values compared to cognitively healthy individuals^40,41^. Our findings support these studies by demonstrating that SSE was predicted by the FAB score (*i.e.* executive function; *β* = 0.282, *p* = 0.014), but not by the MMSE score (*i.e.* learning/memory; *β* = 0.020, *p* = 0.992), which is associated with theta oscillations^22,34^. This suggests that the association between SSE and FAB was affected by changes in higher frequency activities, such as alpha and beta. Alpha is not a single neural oscillatory activity; there are two or more alpha oscillations with distinctive cortical distributions and peak frequencies^42^. A previous study showed that non-pharmacological treatment improved cognitive function in patients with dementia, accompanied by a reduction in alpha oscillatory activity in the temporal cortex^23^. This suggests that larger temporal alpha oscillations are associated with lower values of SSE and severe cognitive dysfunction. Beta activity changes along with healthy ageing^43^; it attenuates in the early stage of AD^44^, and its power in the right frontal cortex is positively correlated with MMSE scores from patients with MCI^45^. A larger beta power predicted a more positive outcome of non-pharmacological treatments^45^, and non-pharmacological treatments enhance beta power in patients with dementia^23^. These results indicate that a reduction in beta oscillations can be associated with lower SSE values. Both alpha and beta oscillations are associated with attention^24,25,46^, working memory^47,48^, and inhibition^25,47,49^, which are all part of executive functions. We posit that changes in high-frequency oscillations (*e.g.* alpha and beta) mediate SSE and FAB.

Regression analyses also showed that the MMSE scores were predicted by age and MF, but not by IAF or SSE (Table 3 and Fig. 3), whereas the FAB scores were predicted only by age, but not by any of the MEG spectral parameters. The asymmetrical relationship between neuropsychological assessment scores and MEG spectral parameters can be explained by the role of age. Correlation analyses showed that age influenced neuropsychological assessment scores more than MEG spectral parameters (Table 1). It is assumed that age explained a large amount of variance in the LMEM predicting neuropsychological assesment scores, which left little room for MEG spectral parameters to contribute to the model (Table 3).

Analyses for all MEG sensors were replicated using MEG SOIs, namely MEG analysis using only frontal sensors (Fnt) or temporal sensors (Tmp), instead of using all 160 sensors, with results demonstrating minor differences [see Supplementary information (Regression analysis with MEG sensors of interest)]. When predicting MEG spectral parameters at SOIs using neuropsychological assesement scores, MF and IAF in both SOIs were predicted by MMSE scores, except TmpMF, whose significance did not meet the threshold after FDR correction (*p* = 0.054) (Supplementary Fig. S3 and Table S5). Further, MF and IAF in both SOIs were not predicted by FAB scores, although MF (for all MEG sensors) was predicted by both MMSE and FAB scores (Fig. 3). There are two possible explanations for this discrepancy. The first is the difference in signal to noise ratio. In terms of the standardised estimated coefficient of the LMEM, although the association of FAB scores with MF and IAF (for all MEG sensors) were significant (MF: *β* = 0.214; IAF: *β* = 0.185), the association of MMSE scores with the same was stronger (MF: *β* = 0.298; IAF: *β* = 0.291). A reduced number of sensors averaged within frontal (35) and temporal (42) SOIs failed to find statistically significant relationships. The second is the contribution of the other 83 MEG sensors, which are neither in Fnt nor Tmp SOIs. If these 83 sensors had any relationship with FAB scores, it would explain that their rejection in the SOI analysis weakens the contributions of FAB scores to the MEG spectral parameters.

Considering the other direction of causality, namely, predicting neuropsychological assesement scores using MEG spectral parameters, frontal and temporal sensors behaved differently (Supplementary Fig. S3 and Table S6). Although age influenced both MMSE and FAB scores in all MEG sensor analyses (“Regression analyses” section, Fig. 3, and Table 3) and FAB in SOI analysis, MMSE was not predicted by age in the SOI analysis (Supplementary Fig. S3 and Table S6). This is consistent with a previous review describing that cognitive impairments associated with the temporal lobe arise from the pathology of AD, while impairments associated with the frontal lobe occur during healthy ageing^50^. The former contributes to changes in MMSE (*i.e.* learning/memory), while the latter contributes to changes in FAB (*i.e.* executive function). The difference in the contribution of age between the two regions led to this discrepancy. The MMSE score was only predicted by TmpSSE, but not by age or the rest of the MEG spectral parameters (Supplementary Fig. S3). The SSE reflects changes in the neural oscillatory activity at any frequency. Temporal delta, theta, alpha, and beta oscillations are associated with MMSE scores^23,51^. Hence, it is reasonable to conclude that TmpSSE influenced the MMSE score. Taken together, the relationships between MMSE scores (*i.e.* learning/memory) and MF/IAF were evident, while the FAB score (*i.e*. executive function) was associated only with SSE.

Regression analysis for individuals with healthy ageing [see Supplementary information (Regression analysis with individuals with healthy ageing)] did not show significant associations between MEG spectral parameters and neuropsychological assessments, which was evident for the whole database (“Regression analyses” section and Tables 2 and 3). The discrepancy can be interpreted in three ways. First, the significant associations were only limited to the pathological conditions. The neuropsychological assessment scores declined for two reasons: healthy ageing^52,53^ and pathological backgrounds, such as Alzheimer's disease^54^. When both sources of variability co-exist (using all 207 individuals), the relationships between MEG parameters and neuropsychological assessment scores were significant; however, when the source of the variability was limited to healthy ageing alone, the relationships did not survive. Previous studies show that resting-state brain activity is sensitive to pathological changes^55,56^. These indicate that MEG parameters are mainly sensitive to a decline due to the pathological changes rather than healthy ageing. Second, the sample size of the dataset was reduced from 207 in the main analysis to 34 in the control analyses. The reduction in the number of examined individuals may have weakened the detection power of the group-level analysis, leading to missed associations between parameters. Third, the ceiling effect of the neuropsychological parameters^57^ reduced the variability of the dataset, which impacted their associations to MEG spectral parameters. Even under the limited conditions, FAB was significantly predicted by age. This result supports the findings of a previous study^58^, which posited that healthy ageing influences cognitive decline as well as the pathological changes.

This study has some limitations. First, the MMSE is not a neuropsychological assessment used to evaluate learning/memory function selectively. However, this did not affect our conclusions because we employed the MMSE as it is not sensitive to executive dysfunction^10^, while the FAB is. The results demonstrated that MMSE and FAB scores mismatched for 59 individuals (Fig. 1), supporting the assumption that MMSE and FAB reflect different sub-domains of cognitive performance, albeit with some potential overlap.

Second, we used three MEG spectral parameters without considering the cortical sources of MEG signals (*i.e.* the analyses were performed at the sensor-level but not at the source-level) because we validated their performance as potential biomarkers in the same way as conducted in clinical practice. Small computational resources (less than 10 min) and simplicity are essential features of clinical biomarkers, making sensor-level analysis outweigh source-level analysis. In addition, our previous study also supports the consistency of the chosen spectral parameters when they are computed at the source- or sensor-level^59^.

Third, this study used clinical records from individuals who had visited the outpatient department for dementia or received medical health check services. Few patients with severe dementia were included; thus, these findings might not reflect a wide range of pathological mechanisms pertinent to dementia. However, the goal of this study was to reveal the sensitivity of potential biomarkers to the two sub-domains of cognitive impairment for screening purposes. Hence, this limitation does not matter in practice.

Fourth, we did not examine classification performance on clinical categories (healthy ageing, MCI, and dementia) using MEG spectral parameters. We acknowledge that the MEG spectral parameters and neuropsychological assesment scores (*i.e.* MMSE and FAB) show no major differences between the clinical categories (between individuals with healthy ageing and MCI)(Supplementary Fig. S1). This indicates that the MEG spectral parameters have a closer relationship with neuropsychological assesment scores than clinical diagnoses. Notably, the relationship between neuropsychological assesment scores and clinical diagnosis is not that simple^60^: an individual with an MMSE score of 30/30 can be diagnosed with dementia^61^. The diagnosis cannot be made using only neuropsychological assesment scores; the MEG spectral parameters are similar. We will address this topic with additional MEG spectral parameters to obtain a comprehensive characterisation of brain dynamics as the next step of our study series.

In conclusion, MEG spectral parameters reflect cognitive impairments not only in the learning/memory domain but also in executive function, which is sometimes affected in the earlier stages of cognitive impairment than learning/memory. Furthermore, this study also provides evidence for the application of MEG spectral parameters as clinical biomarkers of cognitive impairment, such as MCI and dementia.

## Methods

### Participants

We obtained neuropsychological (*i.e.* cognitive) assessment scores and resting-state MEG data retrospectively from 207 individuals (119 women; mean age ± SD: 77.5 ± 8.0 years old, range 43–94 years). They had visited the outpatient department of dementia or received medical health check services and underwent both MMSE/FAB assessments and MEG scans at the Kumagaya General Hospital between 1 April 2019 and 4 August 2021. All of them consented to their data being used for research. Regarding the clinical category, 34 were cognitively healthy individuals, 53 suffered from mild cognitive impairment, and 120 were diagnosed with dementia. Diagnoses were made by a neurosurgeon who was a clinical instructor at the Japanese Society of Dementia Research. The study was approved by the ethics committee of Kumagaya General Hospital (#25 and #40). All methods were performed in accordance with the relevant guidelines and regulations in Japan. All individuals provided written informed consent for participation in this study if they were cognitively healthy. If not, their caregivers provided consent on their behalf.

### Neuropsychological assessments

Cognitive performance was assessed using the Japanese versions of the MMSE and FAB, administrated by clinical psychologists as a part of clinical practice. MMSE and FAB are scored on a scale of 0–30 and 0–18, respectively; lower scores indicate more severe cognitive impairment for both. Here, we defined participants who scored lower than the cut-off of the MMSE (*i.e.* 26 or below)^53^ as MMSE-positive (Mp) and participants who scored lower than the cut-off of the FAB (*i.e.* 12 or below)^62^ as FAB-positive (Fp); otherwise, participants were grouped as MMSE-negative (Mn) and FAB-negative (Fn).

### MEG scanning

Five minutes of resting-state brain activity (*i.e.* spontaneous neural oscillatory activity) was recorded using a 160-channel whole-head type magnetoencephalography system (RICOH160-1; RICOH, Tokyo, Japan) in a magnetically shielded room at Kumagaya General Hospital as a part of clinical practice. During the scan, participants were asked to remain calm in the supine position with their eyes closed. The sampling frequency was 2000 Hz with 500 Hz low-pass filtering during the recording.

### MEG analysis

We applied the same sensor-level analysis protocol used in our previous study^45^ during daily clinical practice at Kumagaya General Hospital. MEG data are sometimes contaminated by artefacts such as signal fluctuation caused by dental works and can deteriorate the quality of analysis. Before starting the detailed analyses, artefacts were manually removed using principal component analysis, if necessary, by experienced clinicians or clinical laboratory technicians. The technique was applied for 41 out of 207 MEG datasets as a part of the clinical practice. The number of removed components were adjusted for each dataset and limited to as few as possible. It was conducted using an analysis software provided by the MEG manufacturer^31,56^,which is authorised for clinical use by the Ministry of Health, Labour, and Welfare of Japan (equivalent to FDA approval). A 50-Hz band-stop filter was applied to remove the power line noise. After removing the artefacts, all MEG analyses were performed offline using MATLAB (MathWorks, MA, USA). Three spectral parameters were calculated to summarise the different properties of spontaneous neural oscillations: MF, IAF, and SSE^41^. Their definitions and details can be found in our previous study^19^. The first parameter, MF, quantifies the frequency at which the spectral power is balanced between low and high frequencies. The frequency divides PSDn into two equal halves between 1 and 70 Hz. The second parameter, IAF, represents the dominant frequency corresponding to the peak of the PSDn in the alpha band (*i.e.* the dominant alpha oscillation). It is defined similarly to MF, but adjusting the frequency range between 4 and 15 Hz (*i.e.* extended alpha band) to obtain a robust estimator of the dominant alpha oscillations^19^. The last parameter, SSE, is defined by applying the definition of the normalised Shannon entropy to the PSDn, which can be assimilated as a probability density function^19^:

$$ {\text{SSE }} = - \frac{1}{log\left( N \right)} \cdot \mathop \sum \limits_{{f = 1\;{\text{Hz}}}}^{{70\;{\text{Hz}}}} PSDn\left( f \right){\text{log}}\left[ {PSDn\left( f \right)} \right] $$

where *N* is the number of frequency bins of the PSDn. SSE represents an irregularity measure closely related to the concept of order in information theory, which quantifies the homogeneity in the distribution of the oscillatory components of the PSDn. Sensor- and epoch-wise MF, IAF, and SSE were computed, then they were averaged across all sensors and epochs. They were referred to without any prefixes such as MF, IAF, and SSE, respectively. Additionally, to investigate the potential effects of the spatial location of sensors, the parameters were averaged within two groups of SOIs instead of whole sensors: frontal (Fnt) and temporal (Tmp) sensors (see Supplementary Fig. S2). The SOI data were used only as supplementary for LMEM analysis.

### Statistical analysis

For clinical verification of data quality and enhancing the comprehensibility of the dataset, descriptive statistics were performed and basic group-level differences were examined [see Supplementary information (Descriptive Statistics)]. Next, correlation analyses were performed using a non-parametric bootstrapping approach to examine the relationships between the MEG spectral parameters and neuropsychological assessments. Bootstrapping statistics have methodological advantages over classical statistical inference (*e.g.* the Gaussian assumption^63^). Correlations were examined among each pair of variables (MF, IAF, SSE, MMSE, and FAB) and participants’ ages. For each pair, Pearson’s coefficient was calculated by resampling with replacement data across all individuals 20,000 times. The percentage of the resampled coefficients, when larger or smaller than 0 (the smaller value), was taken as the significance level (*p*-value). We reported the grand average of the correlation coefficient (*r*) across bootstrap iterations and *p*-values.

Next, the directional relationships between the MEG spectral parameters and neuropsychological assessments were studied using LMEM. The LMEM analysis was performed bidirectionally; contributions of MEG spectral parameters (predictor variables) on neuropsychological assessments (response variables) were studied, and vice versa. For each LMEM, the response variable was subjected to the regression model with three or two fixed covariates (MF, IAF, and SSE when taking neuropsychological scores as response variables; and MMSE and FAB when taking MEG parameters) with an additional fixed covariate: age. To consider sex differences in the model, a random intercept and random slopes (for all fixed predictors) were entered into the model for each sex. The model was estimated using the maximum-likelihood method. Estimated coefficients of fixed predictors were subjected to a *t*-test to check for the null hypothesis that the coefficients would be equal to zero.

As the analyses were exploratory and generated matrices (Tables 1, 2, 3) where each of the statistical values were tested against our null hypothesis (that the coefficients would be equal to zero), this series of results was at risk of an increasing Type-I error^64^. To manage this risk, we reported *p*-values controlled for the false discovery rate (FDR) using the Benjamini–Hochberg method^65^. Significant values are marked with asterisks in all the tables. All statistical analyses were performed using the statistics and machine learning toolbox and multiple testing toolbox^66^ in the MATLAB software.

### Ethics approval and consent to participate

The use of clinical data for the present study was approved by the ethics committee of Kumagaya General Hospital (#25 and #40). Additionally, written informed consent was obtained from the participants or their family members.

## Supplementary Information

Supplementary Information.

## Data Availability

All data generated or analysed during this study are available from Shigihara, Yoshihito (2021), ‘MMSE and FAB study 2021’, Mendeley Data, V1, https://doi.org/10.17632/b7bx8b52nx.1.
