# Supplementary material for: Distinctive effects of executive dysfunction and loss of learning/memory abilities on resting-state brain activity
Source: Sci Rep. 2022 Mar 2;12:3459. doi: 10.1038/s41598-022-07202-7 (PMC8891272; doi:10.1038/s41598-022-07202-7)
Supplement: Supplementary file 1 — Supplementary Information. [file 41598_2022_7202_MOESM1_ESM.pdf]

Supplementary Information

Distinctive effects of executive dysfunction and loss of learning/memory abilities on resting-state brain activity

Hideyuki Hoshi[1], Yoko Hirata[2], Momoko Kobayashi[3], Yuki Sakamoto[3], Keisuke Fukasawa[4], Sayuri Ichikawa[4], Jesús Poza[5,6,7], Víctor Rodríguez-González[5,6], Carlos Gómez[5,6], Yoshihito Shigihara[1,8*]

[1]Precision Medicine Centre, Hokuto Hospital, Japan
[2]Department of neurosurgery, Kumagaya General Hospital, Japan
[3]Precision Medicine Centre, Kumagaya General Hospital, Japan
[4]Clinical Laboratory, Kumagaya General Hospital, Japan
[5]Biomedical Engineering Group, Higher Technical School of Telecommunications Engineering, University of Valladolid, Valladolid, Castilla y León 47011, Spain
[6]Centro de Investigación Biomédica en Red en Bioingeniería, Biomateriales y Nanomedicina, (CIBER-BBN), Valladolid, Castilla y León 47011, Spain
[7]Instituto de Investigación en Matemáticas (IMUVA), University of Valladolid, Valladolid, Castilla y León 47011, Spain
[8]Precision Medicine Centre, Kumagaya General Hospital, Japan

*Correspondence:
Yoshihito Shigihara
Precision Medicine Centre, Hokuto Hospital
Kisen-7-5 Inadacho, Obihiro, Hokkaido, 080-0833, Japan
Tel. no.: +81-155-48-8000
Fax no.: +81-155-48-8000
E-mail: y-shigihara@hokuto7.or.jp

Descriptive Statistics

For clinical verification of data quality and enhancing the comprehensibility of the dataset, descriptive statistics of age, neuropsychological scores [Mini-Mental State Examination (MMSE) and Frontal Assessment Battery (FAB)], and magnetoencephalography (MEG) spectral parameters [median frequency (MF), individual alpha frequency (IAF), and Shannon's spectral entropy (SSE)] were shown for each group [MMSE negative and FAB negative (MnFn), MMSE positive and FAB negative (MpFn), MMSE negative and FAB positive (MnFp), and MMSE positive and FAB positive (MpFp)](Supplementary Fig. S1a and Table S1) and for each clinical category (individuals with healthy ageing, HA; mild cognitive impairment, MCI; and dementia, DEM) (Supplementary Fig. S1b and Table S2). Additionally, average parameters were compared between groups and categories using Analysis of Variance (ANOVA). For comparisons between the groups, the 2×2 between-individual design was employed, where MMSE (2 levels: Mn and Mp) and FAB (2 levels: Fn and Fp) conditions were used as independent variables. The interaction term of MMSE and FAB conditions was also examined. For comparisons between the categories, one-way ANOVA was used, where clinical category (3 levels: HA, MCI, and DEM) condition was used as an independent variable. As one or more main effects or interactions were significant in all comparisons, post-hoc comparisons were performed using Tukey's honestly significant difference procedure. The confidence intervals were also saved for visualisations. The ANOVAs and post-hoc tests were performed using the statistics and machine learning toolbox in the MATLAB software.

The results of the 2×2 ANOVAs between groups showed that the main effects of MMSE and FAB were significant when taking all except SSE parameters as dependent variables (Supplementary Table S3). The individuals in positive groups (Mp and Fp) were older, had lower neuropsychological scores (MMSE and FAB), and obtained smaller MEG spectral parameters (MF and IAF) than those in the negative groups (Mn and Fn). For SSE, the main effect of MMSE was significant, but the one of FAB was not (Supplementary Table S3). The interaction term was significant for modelling MMSE and FAB, which was due to the grouping method; the cut-off score of MMSE was used for grouping Mn and Mp, and that of FAB was used for determining Fn and Fp.

The results of one-way ANOVAs between clinical categories showed that the main effect of category (HA, MCI, and DEM) was significant for all parameters (Supplementary Table S4). The post-hoc test revealed that individuals with dementia had lower neuropsychological scores and smaller MEG spectral parameters than the individuals in the other clinical categories (Supplementary Fig. S1b). However, none of the parameters were significantly different between individuals in HA and MCI categories.

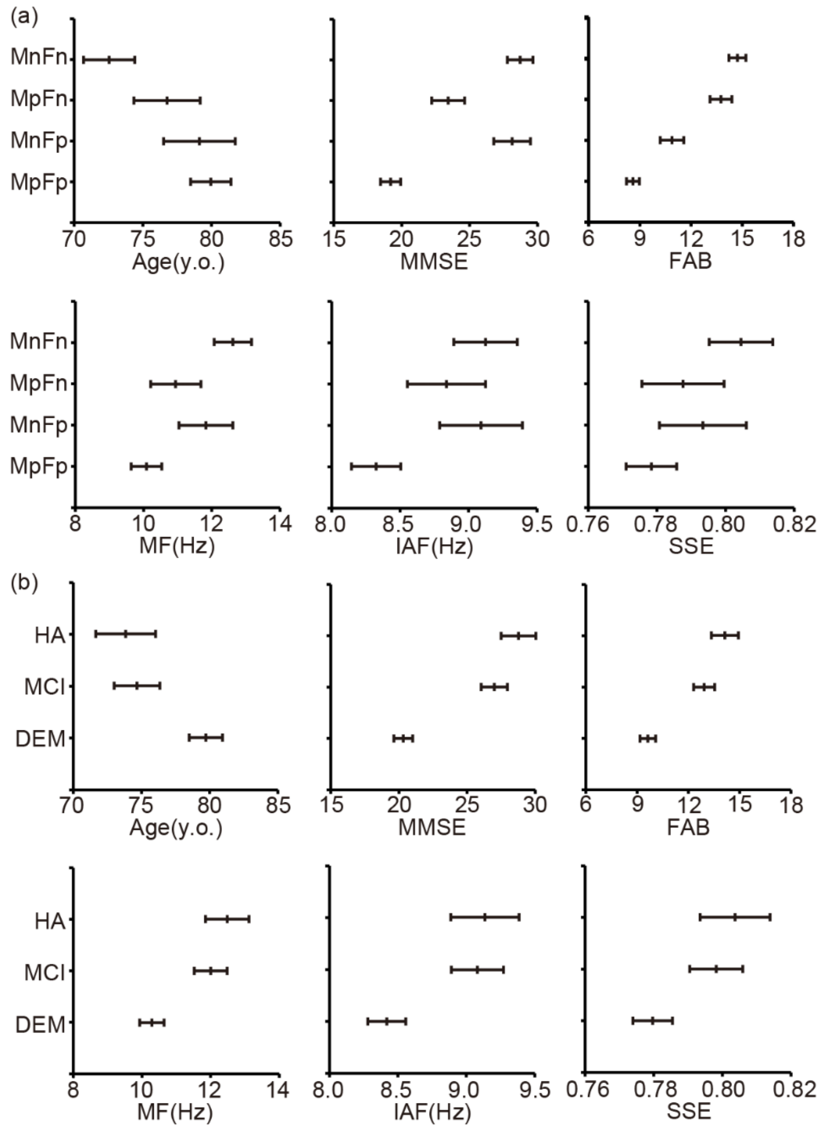

Supplementary Figure S1. Average and comparison interval of age, neuropsychological score, and MEG spectral parameters

In each plot, vertical marks indicate the lower limits, average values, and upper limits of the parameters for (a) each group and (b) each clinical category. The upper and lower limits correspond to the comparison interval of Tukey's honestly significant difference test. Horizontal lines show the comparison intervals computed during the post-hoc test using Tukey's honestly significant difference procedure. MMSE, Mini-Mental State Examination; FAB, Frontal Assessment Battery; MF, Median Frequency; IAF, Individual Alpha Frequency; SSE, Shannon's Spectral Entropy; MnFn, MMSE negative and FAB negative group; MpFn, MMSE positive and FAB negative group; MnFp, MMSE negative and FAB positive group; MpFp, MMSE positive and FAB positive group; HA, individuals in healthy ageing category; MCI, individuals in mild cognitive impairment category; DEM, individuals in dementia category.

Supplementary Table S1. Descriptive statistics (groups)

Group	*N*	Age (y.o)			MMSE			FAB			MF (Hz)			IAF (Hz)			SSE		
		M	±	*SE*	*M*	±	*SE*	*M*	±	*SE*	*M*	±	*SE*	*M*	±	*SE*	*M*	±	*SE*
MnFn	52	72.558	±	1.029	28.750	±	0.511	14.731	±	0.275	12.635	±	0.293	9.126	±	0.117	0.804	±	0.005
MpFn	31	76.742	±	1.333	23.419	±	0.662	13.774	±	0.356	10.929	±	0.380	8.840	±	0.152	0.788	±	0.006
MnFp	28	79.143	±	1.402	28.107	±	0.696	10.893	±	0.374	11.821	±	0.400	9.090	±	0.160	0.793	±	0.007
MpFp	96	79.948	±	0.757	19.188	±	0.376	8.594	±	0.202	10.081	±	0.216	8.322	±	0.086	0.778	±	0.004

N, number of individuals; MMSE, Mini-Mental State Examination; FAB, Frontal Assessment Battery; MF, Median Frequency; IAF, Individual Alpha Frequency; SSE, Shannon's Spectral Entropy; *M*, mean; *SE*, standard error; MnFn, MMSE negative and FAB negative group; MpFn, MMSE positive and FAB negative group; MnFp, MMSE negative and FAB positive group; MpFp, MMSE positive and FAB positive group.

Supplementary Table S2. Descriptive statistics (categories)

Category	*N*	Age (y.o.)			MMSE			FAB			MF (Hz)			IAF (Hz)			SSE		
		M	±	*SE*	*M*	±	*SE*	*M*	±	*SE*	*M*	±	*SE*	*M*	±	*SE*	*M*	±	*SE*
HA	34	73.882	±	1.296	28.765	±	0.738	14.147	±	0.474	12.478	±	0.372	9.136	±	0.147	0.804	±	0.006
MCI	53	74.717	±	1.038	27.000	±	0.591	12.925	±	0.380	12.002	±	0.298	9.082	±	0.118	0.798	±	0.005
DEM	120	79.758	±	0.690	20.342	±	0.393	9.642	±	0.252	10.285	±	0.198	8.417	±	0.078	0.780	±	0.003

N, number of individuals; MMSE, Mini-Mental State Examination; FAB, Frontal Assessment Battery; MF, Median Frequency; IAF, Individual Alpha Frequency; SSE, Shannon's Spectral Entropy; *M*, mean; *SE*, standard error; HA, individuals in healthy ageing category; MCI, individuals in mild cognitive impairment category; DEM, individuals in dementia category.

Supplementary Table S3. Results of ANOVA (groups)

		F	*MS*	*p*
(A) Age	Main effect (MMSE)	4.631	255.001	0.033*
	Main effect (FAB)	17.833	982.040	< 0.001*
	Interaction (MMSE×FAB)	2.124	116.973	0.147
(B) MMSE	Main effect (MMSE)	153.302	2080.221	< 0.001*
	Main effect (FAB)	17.939	243.422	< 0.001*
	Interaction (MMSE×FAB)	9.724	131.950	0.002*
(C) FAB	Main effect (MMSE)	27.708	108.579	< 0.001*
	Main effect (FAB)	212.609	833.138	< 0.001*
	Interaction (MMSE×FAB)	4.712	18.463	0.031*
(D) MF	Main effect (MMSE)	27.185	121.735	< 0.001*
	Main effect (FAB)	6.321	28.307	0.013*
	Interaction (MMSE×FAB)	0.003	0.012	0.959
(E) IAF	Main effect (MMSE)	15.858	11.378	< 0.001*
	Main effect (FAB)	4.368	3.134	0.038*
	Interaction (MMSE×FAB)	3.317	2.380	0.070
(F) SSE	Main effect (MMSE)	8.408	0.010	0.004*
	Main effect (FAB)	3.395	0.004	0.067
	Interaction (MMSE×FAB)	0.026	< 0.001	0.873

F, *F*-statistic; *MS*, Mean squares for each effect; *p*, *p*-values; MMSE, Mini-Mental State Examination; FAB, Frontal Assessment Battery; MF, Median Frequency; IAF, Individual Alpha Frequency; SSE, Shannon's Spectral Entropy. An asterisk (*) indicates significant *p*-values.

Supplementary Table S4. Results of ANOVA (main effect of category)

	F	*MS*	*p*
(A) Age	12.848	733.736	< 0.001*
(B) MMSE	75.239	1393.812	< 0.001*
(C) FAB	48.675	372.112	< 0.001*
(D) MF	19.932	93.733	< 0.001*
(E) IAF	16.186	11.891	< 0.001*
(F) SSE	8.995	0.011	< 0.001*

F, *F*-statistic; *MS*, Mean squares for each effect; *p*, *p*-values; MMSE, Mini-Mental State Examination; FAB, Frontal Assessment Battery; MF, Median Frequency; IAF, Individual Alpha Frequency; SSE, Shannon's Spectral Entropy. An asterisk (*) indicates significant *p*-values.

Regression analysis with MEG sensors of interest (frontal and temporal regions)

The MMSE assesses learning/memory ability[1] which mainly depends on the temporal lobe[2], while the FAB evaluates executive function[3], which depends on the frontal lobe[4]. Therefore, the temporal and frontal sensors may be more sensitive to the MMSE and FAB scores. To examine the possible regional differences in the relationships between these two sub-domains of cognitive performance and MEG spectral parameters, we applied a sensor of interest (SOI) analysis using only frontal (Fnt) and temporal (Tmp) sensors. The SOIs were defined by the MEG manufacturer (Supplementary Fig. S2). The MEG spectral parameters averaged within each SOI were referred to with prefixes corresponding to the channel locations; for example, FntMF refers to MF averaged within frontal sensors.

Results showed that MF and IAF in both SOIs (Fnt and Tmp) were predicted by MMSE alone (FntMF: $\beta = 0.315$, $p = 0.007$; FntIAF: $\beta = 0.295$, $p = 0.012$; TmpIAF: $\beta = 0.274$, $p = 0.013$), except TmpMF ($\beta = 0.227$, $p = 0.054$) (Supplementary Table S5). FntSSE was predicted by FAB ($\beta = 0.265$, $p = 0.032$) alone. No significant predictors were found for TmpSSE. MMSE was predicted by TmpSSE ($\beta = -0.395$, $p = 0.018$), but not by age or any MEG spectral parameter (Supplementary Table S6). FAB was predicted by age alone ($\beta = -0.278$, $p < 0.001$), but not by any MEG spectral parameter.

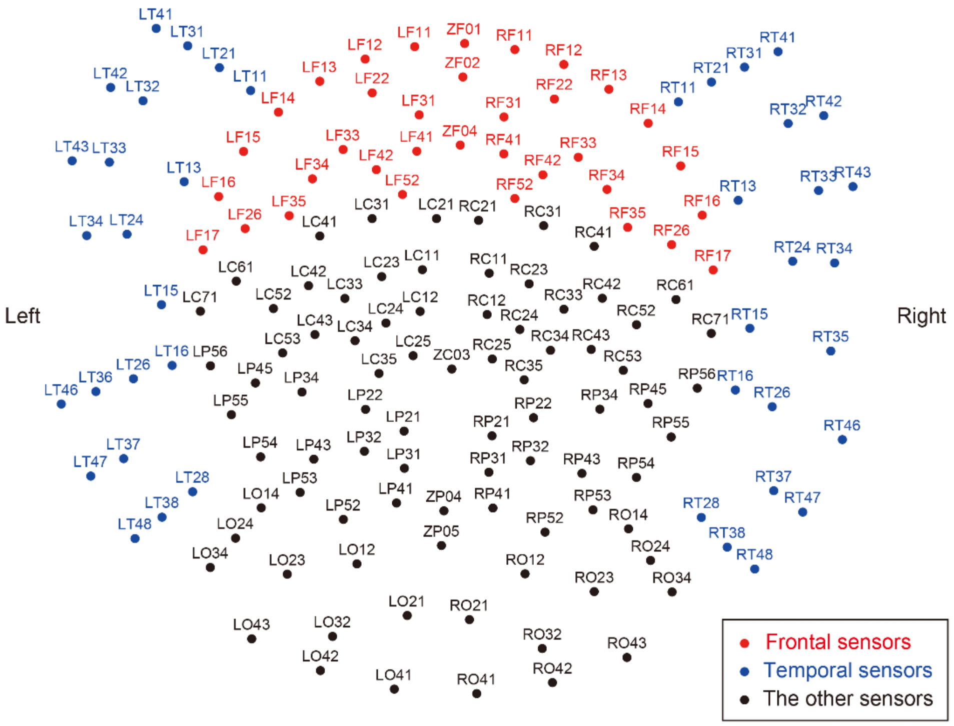

Supplementary Figure S2. MEG sensor layout viewed from above

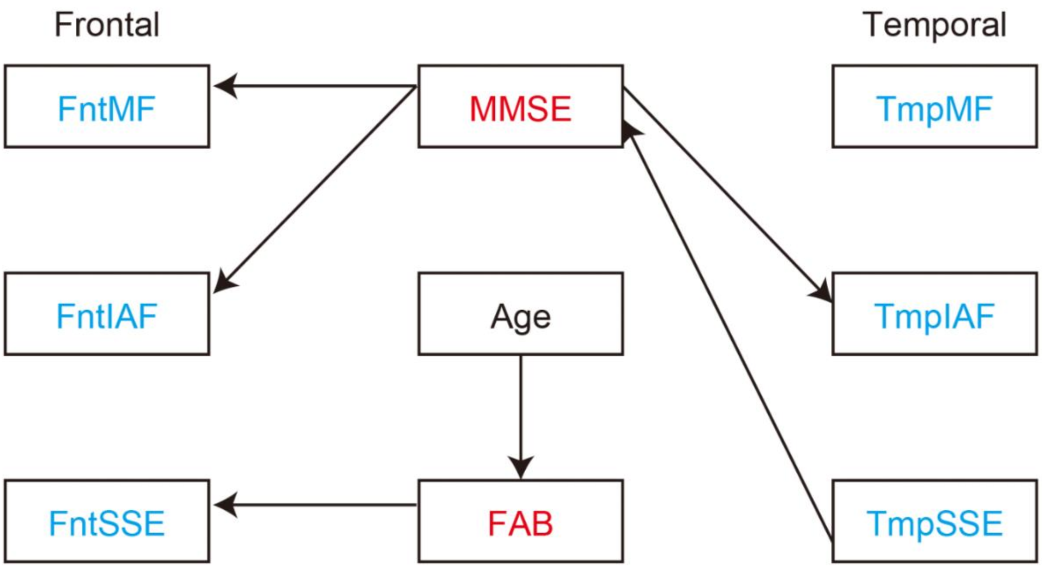

Supplementary Figure S3. Associations between neuropsychological assessment scores, MEG spectral parameters (at sensors of interest), and age

Arrows indicate significant directional influence revealed by regression analysis at sensor of interest. MMSE, Mini-Mental State Examination; FAB, Frontal Assessment Battery; Fnt, frontal sensors; Tmp, temporal sensors; MF, Median Frequency; IAF, Individual Alpha Frequency; SSE, Shannon’s Spectral Entropy.

Supplementary Table S5. Results of LMEM examining the effects of age and neuropsychological assessment scores on MEG spectral parameters (at sensors of interest)

	FntMF				FntIAF				FntSSE			
	β	*SE*	*t*	*p* (FDR)	β	*SE*	*t*	*p* (FDR)	β	*SE*	*t*	*p* (FDR)
Intercept	-0.015	0.088	-0.170	0.989	-0.013	0.071	-0.179	0.989	< 0.001	0.066	-0.002	0.999
Age	0.031	0.079	0.390	0.989	0.051	0.098	0.521	0.965	-0.007	0.071	-0.096	0.999
MMSE	0.315	0.086	3.670	0.007*	0.295	0.089	3.332	0.012*	0.113	0.091	1.245	0.469
FAB	0.175	0.089	1.962	0.164	0.132	0.092	1.429	0.403	0.265	0.094	2.818	0.032*

MMSE, Mini-Mental State Examination; FAB, Frontal Assessment Battery; Fnt, frontal sensors; Tmp, temporal sensors; MF, Median Frequency; IAF, Individual Alpha Frequency; SSE, Shannon's Spectral Entropy; β, standardised estimated coefficient of the predictor; *t*, *t*-values for testing the null hypothesis that the coefficients are equal to zero; *SE*, standard error of estimated coefficient; *p* (FDR), *p*-values of *t*-test with FDR correction. An asterisk (*) indicates terms that make significant contributions to the model.

Supplementary Table S5. (Cont.)

	TmpMF				TmpIAF				TmpSSE			
	β	*SE*	*t*	*p* (FDR)	β	*SE*	*t*	*p* (FDR)	β	*SE*	*t*	*p* (FDR)
Intercept	-0.021	0.117	-0.178	0.989	-0.019	0.092	-0.203	0.989	< 0.001	0.084	0.002	0.999
Age	-0.066	0.069	-0.948	0.688	-0.075	0.089	-0.841	0.688	-0.031	0.107	-0.291	0.989
MMSE	0.227	0.089	2.543	0.054	0.274	0.086	3.202	0.013*	-0.081	0.094	-0.855	0.688
FAB	0.132	0.095	1.384	0.403	0.172	0.089	1.932	0.164	0.243	0.098	2.491	0.054

Supplementary Table S6. Results of LMEM examining the effects of age and MEG spectral parameters (at sensors of interest) on neuropsychological assessment scores

		MMSE				FAB			
		β	*SE*	*t*	*p* (FDR)	β	*SE*	*t*	*p* (FDR)
Intercept		0.013	0.064	0.201	0.961	-0.004	0.082	-0.049	0.986
Age		-0.156	0.061	-2.554	0.061	-0.278	0.061	-4.543	< 0.001*
Fnt	MF	0.335	0.174	1.920	0.129	0.269	0.154	1.752	0.163
	IAF	-0.212	0.151	-1.409	0.246	-0.208	0.151	-1.381	0.246
	SSE	0.309	0.140	2.202	0.092	0.231	0.141	1.643	0.181
Tmp	MF	-0.003	0.152	-0.017	0.986	-0.134	0.152	-0.880	0.467
	IAF	0.336	0.167	2.010	0.122	0.380	0.168	2.267	0.092
	SSE	-0.395	0.128	-3.089	0.018*	-0.157	0.128	-1.229	0.294

Fnt, frontal sensors; Tmp, temporal sensors; MF, Median Frequency; IAF, Individual Alpha Frequency; SSE, Shannon's Spectral Entropy; MMSE, Mini-Mental State Examination; FAB, Frontal Assessment Battery; β, estimated coefficient of the predictor; *t*, *t*-values for testing the null hypothesis that the coefficients are equal to zero; *SE*, standard error of estimated coefficient; *p* (FDR), *p*-values of *t*-test with FDR correction. An asterisk (*) indicates terms that make significant contributions to the model.

Regression analysis with individuals with healthy ageing (Control analyses)

Thirty-four out of the 207 individuals in the dataset were individuals with HA. To examine whether the associations between MEG spectral parameters and neuropsychological assessments were observed only in the HA category, we carried out a regression analysis similar to the one performed using the complete dataset of 207 individuals. The results showed that none of the MEG spectral parameters predicted neuropsychological assessments and *vice versa* (Supplementary Table S7 and S8). The age, included in the linear mixed-effect model as a nuisance predictor, was the only significant term for predicting FAB score in a negative-fashion ($\beta = -0.544$, $p = 0.009$).

Supplementary Table S7. Results of LMEM examining the effects of age and neuropsychological assessment scores on MEG spectral parameters (using the healthy ageing dataset)

	MF				IAF				SSE			
	β	*SE*	*t*	p (FDR)	β	*SE*	*t*	p (FDR)	β	*SE*	*t*	*p* (FDR)
Intercept	< 0.001	0.156	< 0.001	1.000	< 0.001	0.159	< 0.001	1.000	< 0.001	0.161	< 0.001	1.000
Age	0.017	0.192	0.090	1.000	-0.229	0.195	-1.172	0.660	-0.195	0.197	-0.989	0.660
MMSE	-0.154	0.175	-0.882	0.660	-0.236	0.178	-1.330	0.660	-0.068	0.179	-0.380	1.000
FAB	0.414	0.193	2.145	0.482	0.179	0.196	0.912	0.660	0.187	0.198	0.945	0.660

MMSE, Mini-Mental State Examination; FAB, Frontal Assessment Battery; MF, Median Frequency; IAF, Individual Alpha Frequency; SSE, Shannon's Spectral Entropy; β, standardised estimated coefficient of the predictor; *t*, *t*-values for testing the null hypothesis that the coefficients are equal to zero; *SE*, standard error of estimated coefficient; *p* (FDR), *p*-values of *t*-test with FDR correction.

Supplementary Table S8. Results of LMEM examining the effects of age and MEG spectral parameters on neuropsychological assessment scores (using the healthy ageing dataset)

	MMSE				FAB			
	β	*SE*	*t*	*p* (FDR)	β	*SE*	*t*	*p* (FDR)
Intercept	0.052	0.241	0.214	1.000	< 0.001	0.134	< 0.001	1.000
Age	-0.369	0.162	-2.269	0.155	-0.544	0.147	-3.693	0.009*
MF	0.178	0.255	0.697	0.702	0.454	0.234	1.942	0.206
IAF	-0.243	0.204	-1.189	0.610	-0.165	0.188	-0.878	0.702
SSE	0.025	0.265	0.093	1.000	-0.141	0.196	-0.718	0.702

MF, Median Frequency; IAF, Individual Alpha Frequency; SSE, Shannon's Spectral Entropy; MMSE, Mini-Mental State Examination; FAB, Frontal Assessment Battery; β, estimated coefficient of the predictor; *t*, *t*-values for testing the null hypothesis that the coefficients are equal to zero; *SE*, standard error of estimated coefficient; *p* (FDR), *p*-values of *t*-test with FDR correction. An asterisk (*) indicates terms that make significant contributions to the model.

References

1. Dinomais, M. *et al.* Anatomic correlation of the mini-mental state examination: A voxel-based morphometric study in older adults. *PLoS One* **11**, e0162889 (2016).
2. Jahn, H. Memory loss in Alzheimer's disease. *Dial. Clin. Neurosci.* **15**, 445–454 (2013).
3. Dubois, B., Slachevsky, A., Litvan, I. & Pillon, B. The FAB: A Frontal Assessment Battery at bedside. *Neurology* **55**, 1621–1626 (2000).
4. Gilbert, S. J. & Burgess, P. W. Executive function. *Current Biology* vol. 18 R110–R114 (2008).
5. Murman, D. L. The Impact of Age on Cognition. *Semin. Hear.* **36**, 111–121 (2015).
6. Boban, M. *et al.* The reliability and validity of the mini-mental state examination in the elderly Croatian population. *Dement. Geriatr. Cogn. Disord.* **33**, 385–392 (2012).
7. Budson, A. & Solomon, P. *Memory Loss, Alzheimer's Disease, and Dementia - E-Book: A Practical Guide for Clinicians*. (Elsevier, 2021).
8. Nakamura, A. *et al.* Electromagnetic signatures of the preclinical and prodromal stages of Alzheimer's disease. *Brain* **141**, 1470–1485 (2018).
9. Matsumoto, T. *et al.* The association between carotid blood flow and resting-state brain activity in patients with cerebrovascular diseases. *Sci. Rep.* **11**, 1–11 (2021).
10. Trzepacz, P. T., Hochstetler, H., Wang, S., Walker, B. & Saykin, A. J. Relationship between the Montreal Cognitive Assessment and Mini-mental State Examination for assessment of mild cognitive impairment in older adults. *BMC Geriatr.* **15**, (2015).
11. Coen, R. F. *et al.* The Frontal Assessment Battery: Normative Performance in a Large Sample of Older Community-Dwelling Hospital Outpatient or General Practitioner Attenders. *J. Geriatr. Psychiatry Neurol.* **29**, 338–343 (2016).
